# Supplementary material for: Controlled Synthesis of Up-Conversion NaYF4:Yb,Tm Nanoparticles for Drug Release under Near IR-Light Therapy
Source: Biomedicines. 2021 Dec 20;9(12):1953. doi: 10.3390/biomedicines9121953 (PMC8699023; doi:10.3390/biomedicines9121953)
Supplement: Supplementary file 1 [file biomedicines-09-01953-s001.zip › biomedicines-1486210-supplementary.pdf]

Supplementary Information

Controlled Synthesis of Up-Conversion $NaYF_4$:Yb,Tm Nanoparticles for Drug Release Under Near IR-Light Therapy

Edelweiss Moyano Rodríguez[a,b], Miguel Gomez-Mendoza[b], Raúl Pérez-Ruiz[b,c], Beatriz Peñín[d], Diego Sampedro[d], Antonio Caamaño[a], Víctor A. de la Peña O'Shea[b]

[a] Signal theory and communications and telematic system and computing, Rey Juan Carlos University, Cam/ del Molin 5; 28942 Fuenlabrada, Madrid, Spain.

[b] Photoactivated Processes Unit IMDEA Energy Institute, C/ Ramón de la Sagra, 3 28935 Móstoles, Madrid, Spain.

[c] Departamento de Química, Universitat Politècnica de Valencia, Camno de Vera S/N, 46022, Valencia, Spain.

[d] Departamento de Química, Centro de Investigación en Síntesis Química (CISQ), Universidad de La Rioja, Madre de Dios, 53, 26006, Logroño, Spain.

Table of contents

Scheme S1. Synthetic route for the dyads **CF (1)** and **CF-OE (2)** **S3**

Figure S1. ^{1}H (up) and ^{13}C (down) NMR of **CF-OE 2** **S4**

Figure S2. Mass spectrometer **c**haracterization data of **CF-OE 2** **S5**

Figure S3. TEM images of A) UC1; B) UC2 and; C) UC3 **S6**

Figure S4. TEM images of A) UC4; B) UC5, C) UC6 and; D) UC7. Inset in figure S2C correspond to electron diffraction of red squared area **S7**

Figure S5. Absorption spectra of CF-OE (0.5 mM) in the presence of increasing amounts of formic acid (% v/v). Inset: zoom image. **S8**

Figure S6. A) Transient absorption spectrum (TAS, λ_{exc} = 355 nm) for CF in deaerated dichloromethane solution immediately after the laser pulse and B) upon 100 ns for CF (red), CF-OE (black) or CF-OE un acidic media (blue) in deaerated dichloromethane solutions. Inset: decay trace monitored at 650 nm for CF-OE as model **S9**

Figure S7. A and B) HPLC chromatograms for CF (A) and CF-OE (B) upon increasing concentration in aerated 70:50 H_2O : CH_3CN, containing 0.1% of FA. Inset: corresponding linear calibration curve. C and D) Calibration curve for CF (C) and CF-OE (D). The measurements were performed by triplicated for each compound **S10**

Figure S8. HPLC chromatograms of the evolution formation of free CF (red) and disappearance of CF-OE compound (blue) upon increasing irradiation times (λ_{exc} = 980 nm), at 0.5 M of CF-OE in deaerated methylene chloride (10 % FA v/v). The mobile phase used in HPLC analysis was 70:50 H_2O:CH_3CN, containing 0.1% of FA **S11**

Figure S9. HPLC chromatograms of the formation of the benzophenone (BP) as a second product upon increasing irradiation times (λ_{exc} = 980 nm), at 0.5 mM of CF-OE in deaerated methylene chloride (10 % FA v/v). The mobile phase used in HPLC analysis was 70:50 H2O : CH3CN, containing 0.1% of FA. The orange trace corresponds to the commercial BP reference **S12**

Figure S10. Reusability of UC3 by monitoring the conversion rate of the formation of CF upon increasing irradiation times (λ_{exc} = 980 nm) during five consecutive cycles. Cycle 1 to 5: black, red, blue, green and purple, respectively **S13**

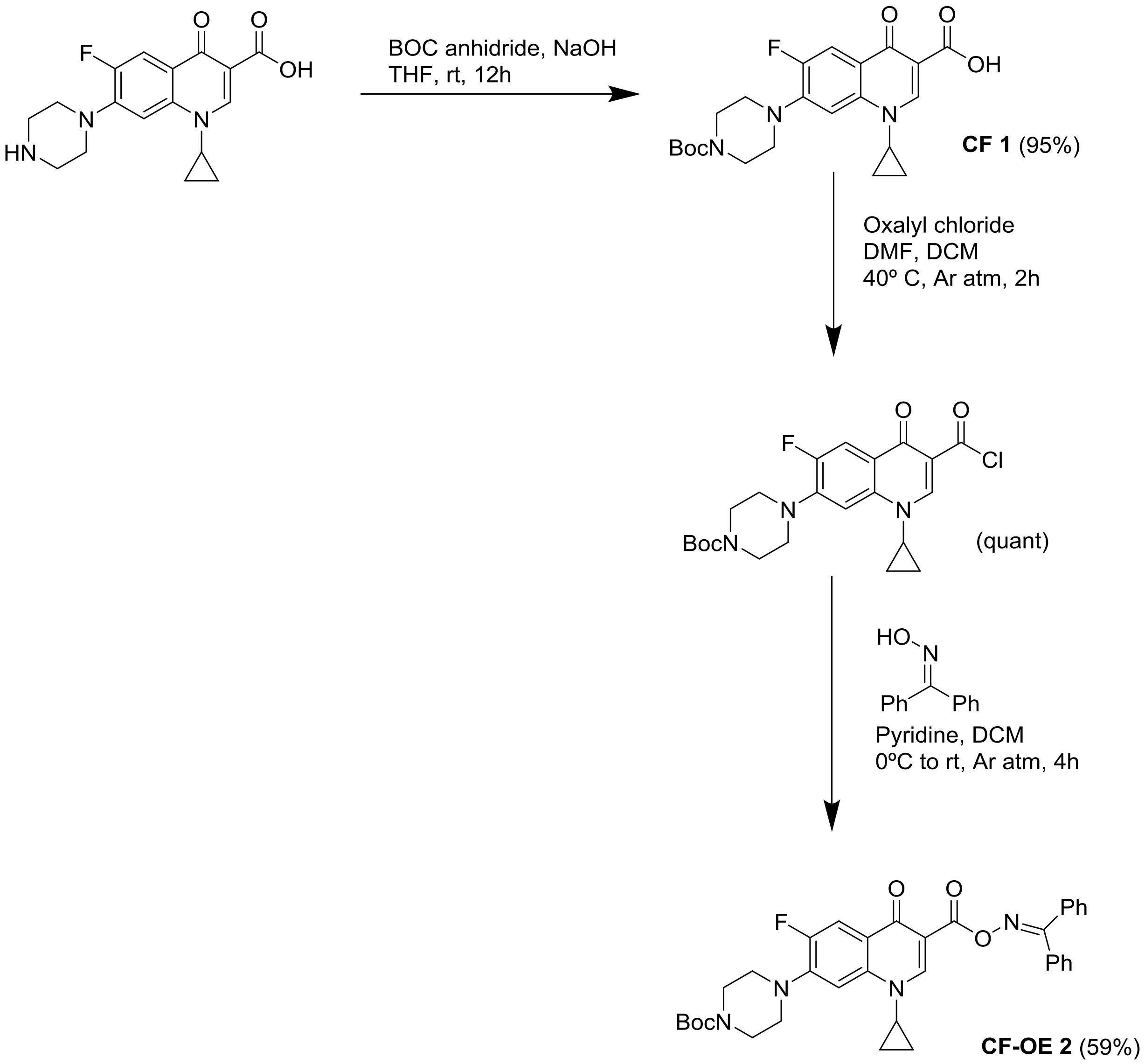

Scheme S1. Synthetic route for the dyads ciprofloxacin **CF 1** and its corresponding oxime-ester derivative **CF-OE 2**.

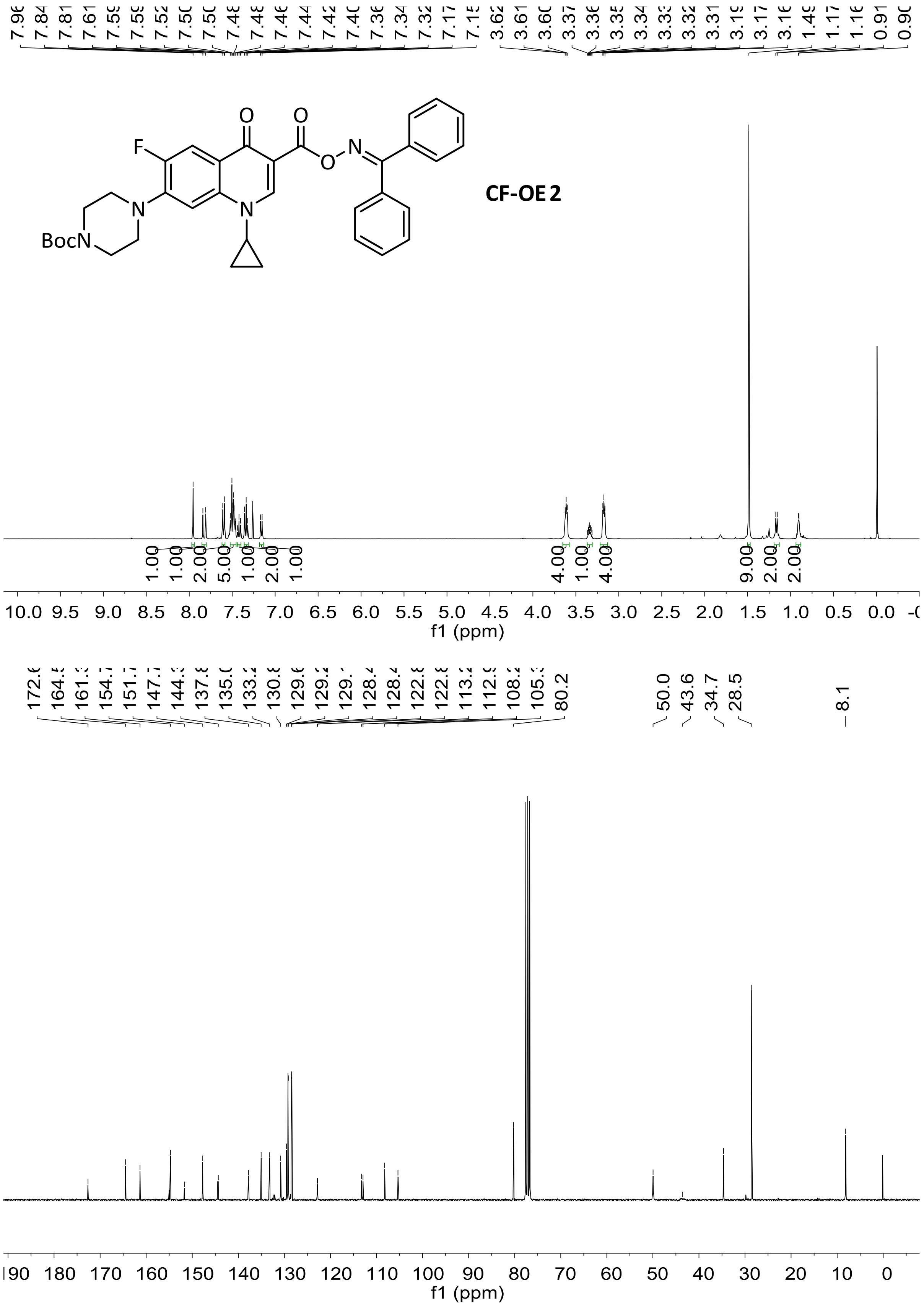

Figure S1. ^{1}H (up) and ^{13}C (down) NMR of **CF-OE 2**

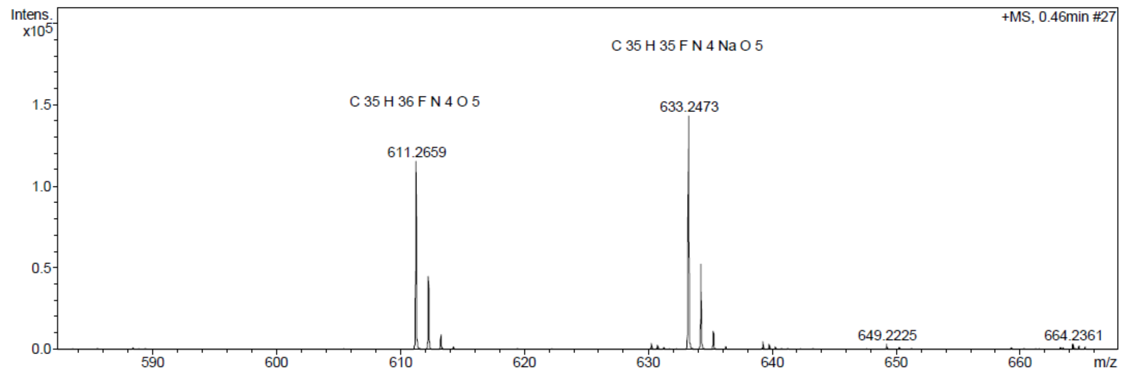

Formula	m/z	z	|err| [ppm]	Mean err [ppm]	mSigma
C 35 H 35 F N 4 Na O 5	633.2484	1+	1.7	2.0	16.5
C 35 H 36 F N 4 O 5	611.2664		0.9	1.3	5.6

Figure S2. Mass spectrometer **c**haracterization data of **CF-OE 2**.

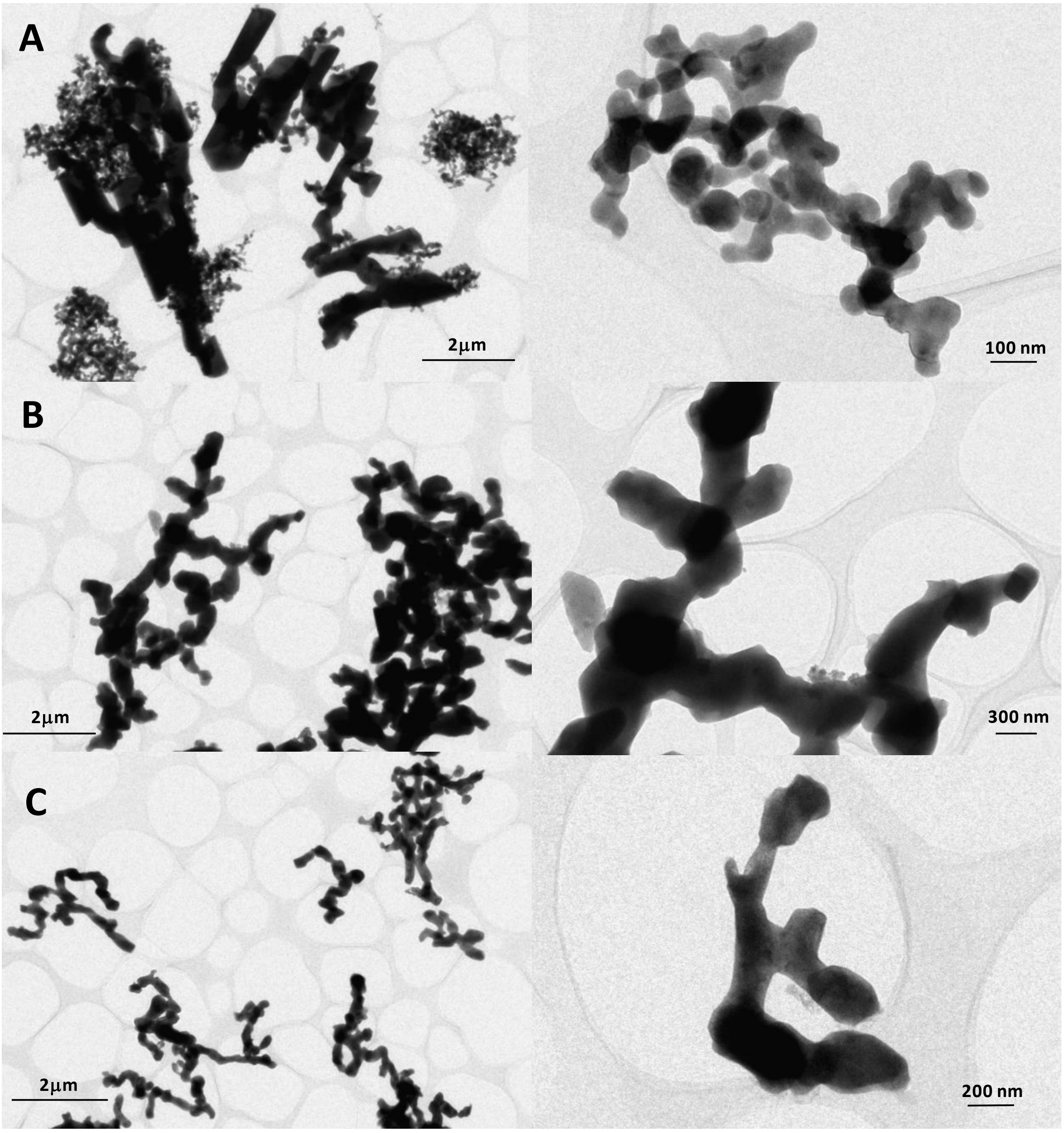

Figure S3. TEM images of A) UC1; B) UC2 and; C) UC3

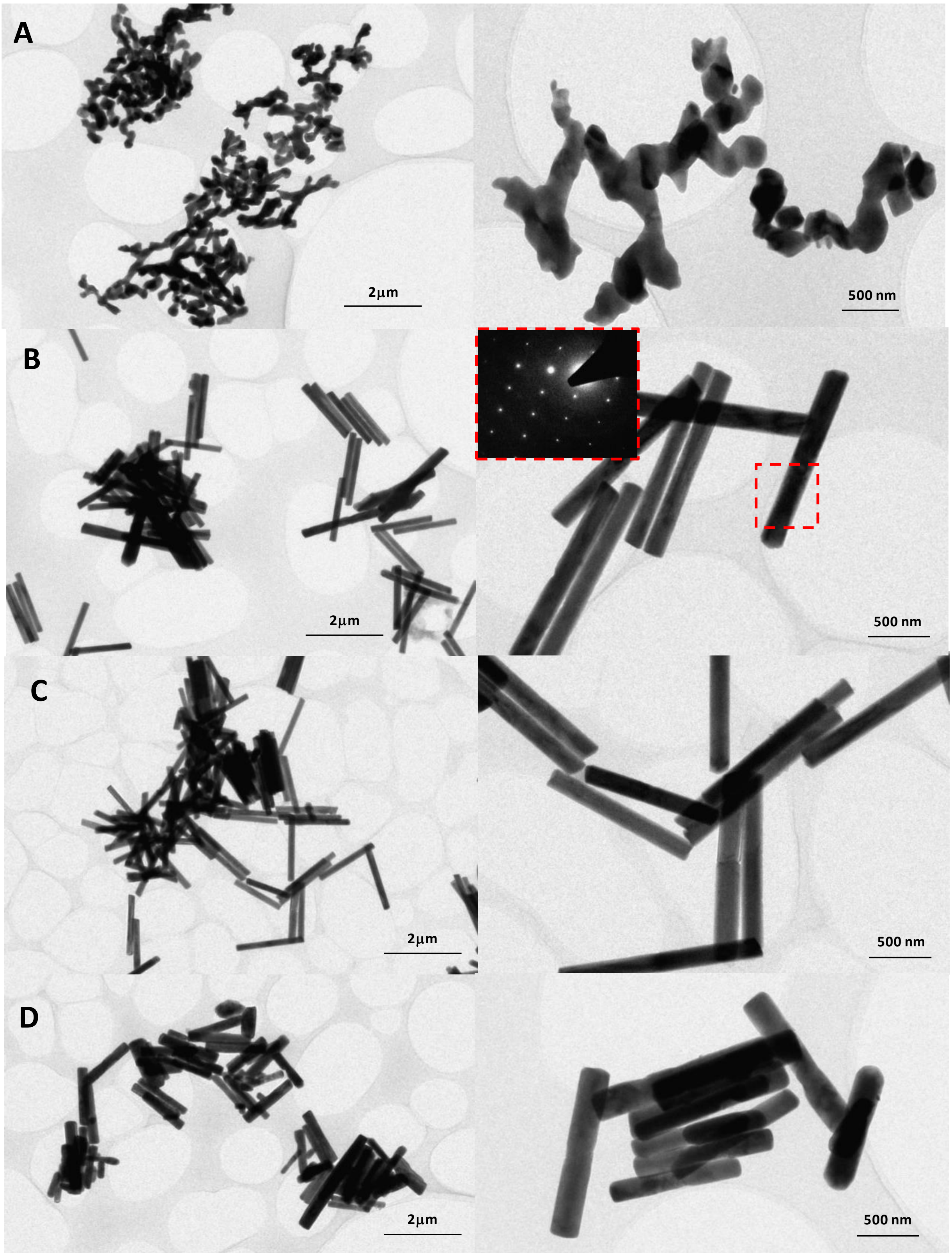

Figure S4. TEM images of A) UC4; B) UC5, C) UC6 and; D) UC7. Inset in figure S2C correspond to electron diffraction of red squared area.

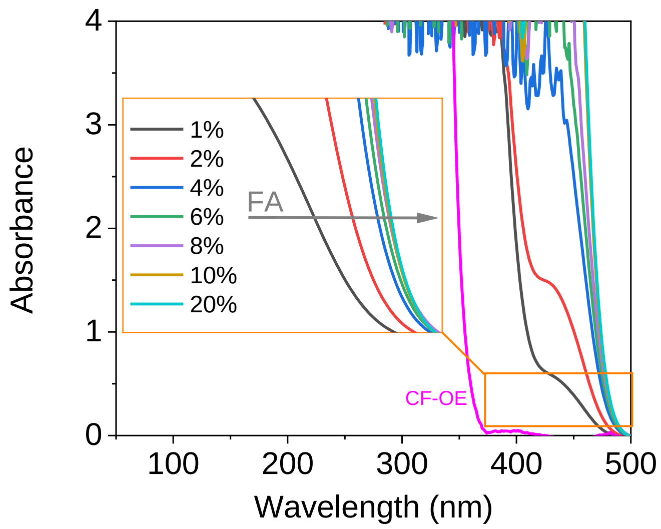

Figure S5. Absorption spectra of CF-OE (pink, 0.5 mM) in the presence of increasing amounts of formic acid (% v/v). Inset: zoom image.

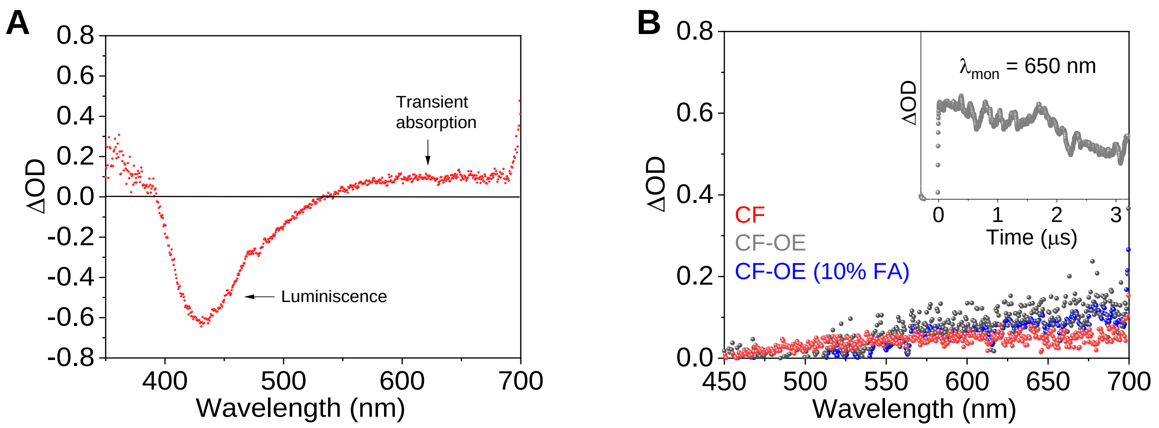

Figure S6. A) Transient absorption spectrum (TAS, λ_{exc} = 355 nm) for CF in deaerated dichloromethane solution immediately after the laser pulse and B) upon 100 ns for CF (red), CF-OE (black) or CF-OE un acidic media (blue) in deaerated dichloromethane solutions. Inset: decay trace monitored at 650 nm for CF-OE as model.

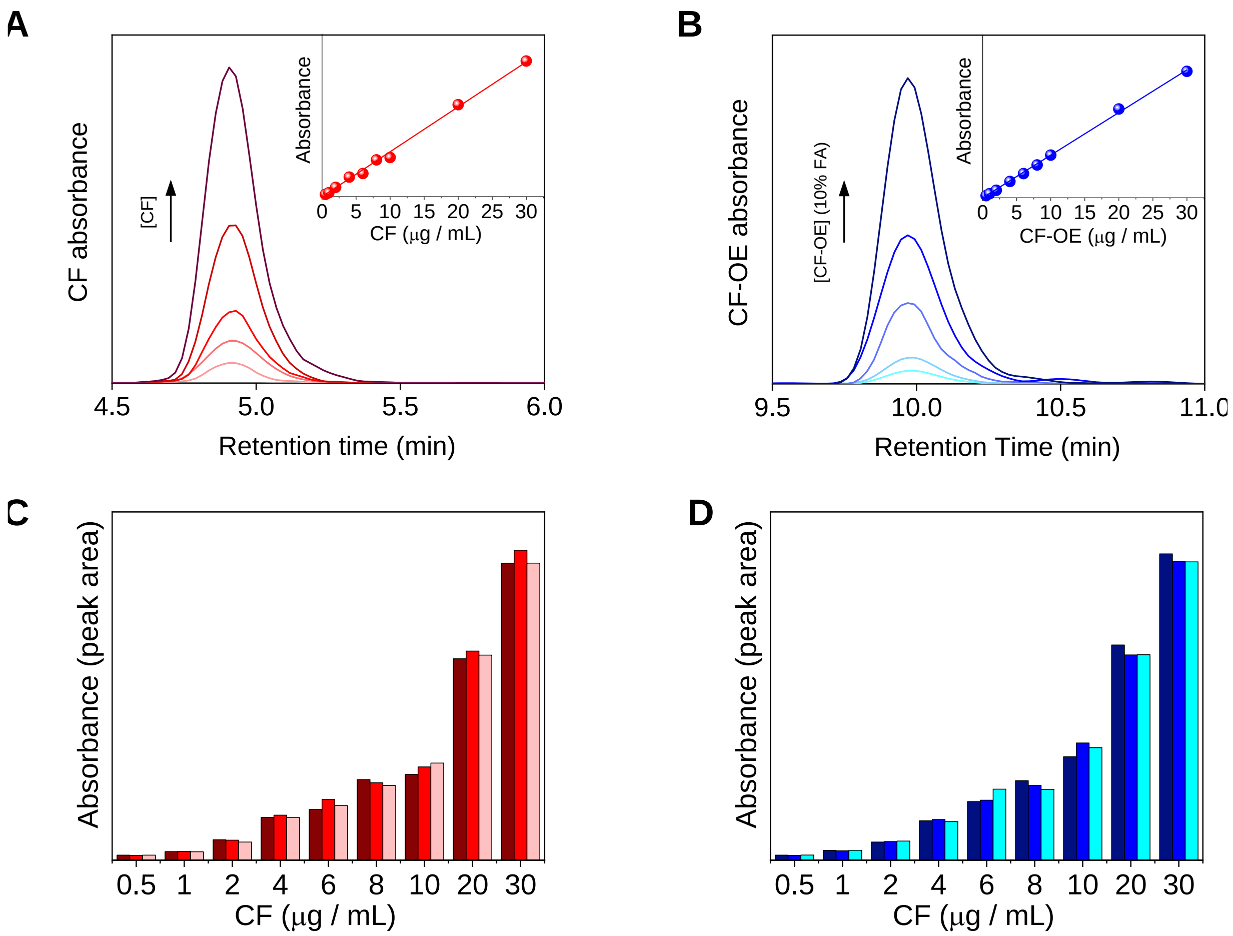

Figure S7. A and B) HPLC chromatograms for CF (A) and CF-OE (B) upon increasing concentration in aerated 70:50 H_2O : CH_3CN, containing 0.1% of FA. Inset: corresponding linear calibration curve. C and D) Calibration curve for CF (C) and CF-OE (D). The measurements were performed by triplicated for each compound.

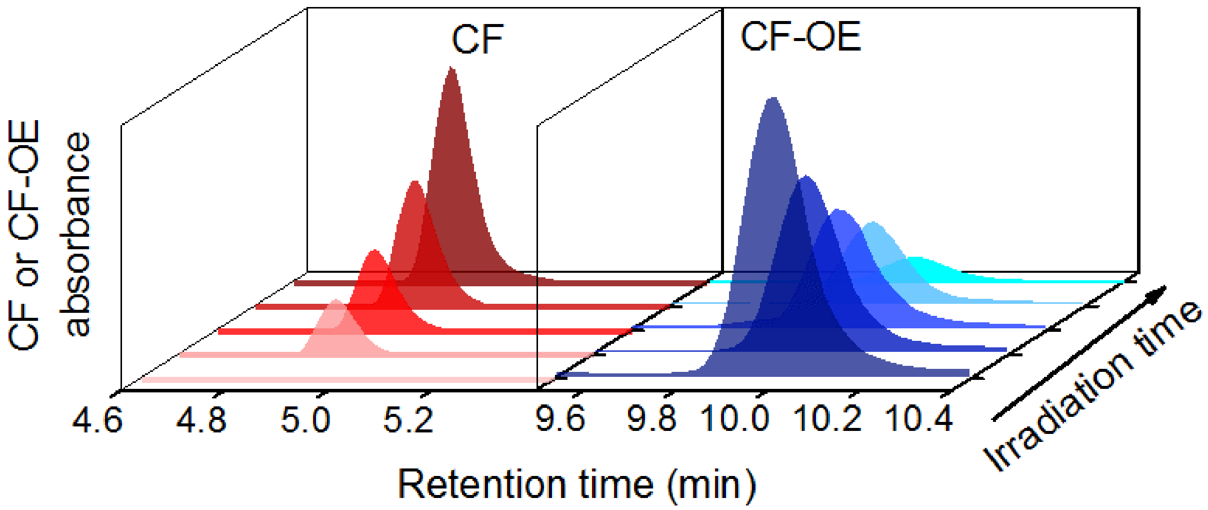

Figure S8. HPLC chromatograms of the evolution formation of free CF (red) and disappearance of CF-OE compound (blue) upon increasing irradiation times (λ_{exc} = 980 nm), at 0.5 M of CF-OE in deaerated methylene chloride (10 % FA v/v). The mobile phase used in HPLC analysis was 70:50 H_2O : CH_3CN, containing 0.1% of FA.

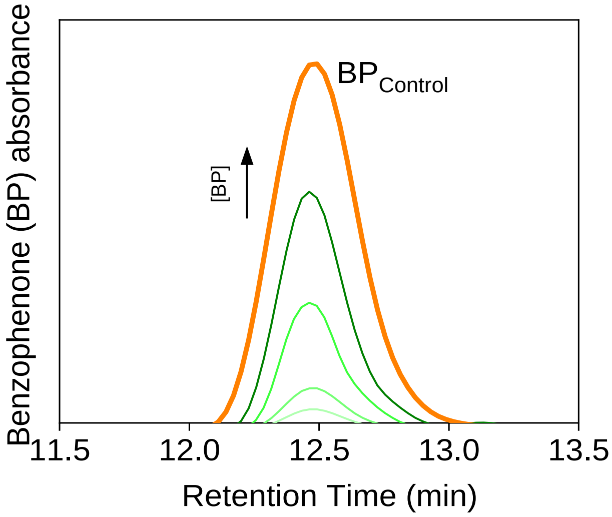

Figure S9. HPLC chromatograms of the formation of the benzophenone (BP) as a second product upon increasing irradiation times (λ_{exc} = 980 nm), at 0.5 mM of CF-OE in deaerated methylene chloride (10 % FA v/v). The mobile phase used in HPLC analysis was 70:50 H_2O : CH_3CN, containing 0.1% of FA. The orange trace corresponds to the commercial BP reference.

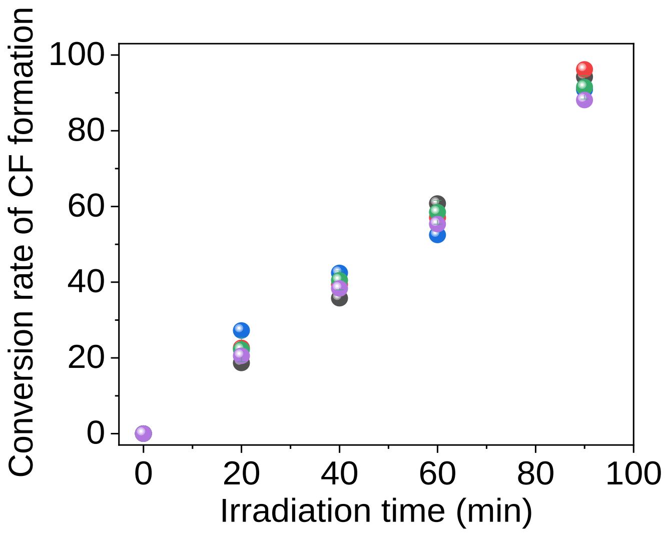

Figure S10. Reusability of UC3 by monitoring the conversion rate of the formation of CF upon increasing irradiation times (λ_{exc} = 980 nm) during five consecutive cycles. Cycle 1 to 5: black, red, blue, green and purple, respectively.
